# Supplementary material for: Influence of flicker noise and nonlinearity on the frequency spectrum of spin torque nano-oscillators
Source: Sci Rep. 2020 Aug 4;10:13116. doi: 10.1038/s41598-020-70076-0 (PMC7403434; doi:10.1038/s41598-020-70076-0)
Supplement: Supplementary file 1 — Supplementary Information. [file 41598_2020_70076_MOESM1_ESM.pdf]

Influence of flicker noise and nonlinearity on the frequency spectrum of spin torque nano-oscillators
# – Supplementary –

Steffen Wittrock,[1,*] Philippe Talatchian,[1,2] Sumito Tsunegi,[3] Denis Crété,[1] Kay Yakushiji,[3] Paolo Bortolotti,[1] Ursula Ebels,[4] Akio Fukushima,[3] Hitoshi Kubota,[3] Shinji Yuasa,[3] Julie Grollier,[1] Gilles Cibiel,[5] Serge Galliou,[6] Enrico Rubiola,[6] and Vincent Cros[1,†]

[1] *Unité Mixte de Physique CNRS/Thales, Univ. Paris-Sud, Univ. Paris-Saclay, 1 Avenue Augustin Fresnel, 91767 Palaiseau, France*
[2] *Institute for Research in Electronics and Applied Physics, Univ. of Maryland, College Park, 20899-6202, MD, USA*
[3] *National Institute of Advanced Industrial Science and Technology (AIST), Spintronics Research Center, Tsukuba, Ibaraki 305-8568, Japan*
[4] *Univ. Grenoble Alpes, CEA, INAC-SPINTEC, CNRS, SPINTEC, 38000 Grenoble, France*
[5] *Centre National d'Études Spatiales (CNES), 18 av. Edouard Belin, 31401 Toulouse, France*
[6] *FEMTO-ST Institute, CNRS, Univ. Bourgogne Franche Comté, 25030 Besançon, France*
(Dated: April 2, 2020)

SUPPLEMENTARY

A. Thiele simulation

The simulation of the vortex dynamics is performed based on the differential Thiele equation[1]. It describes the spin transfer torque iduced gyrotropic motion of the vortex core well. Following the description in Refs.[2,3], we also consider higher order terms of damping and confinement, what allows a deterministic description of the dynamics through the normalized oscillation radius $s(t)$ and phase $\theta(t)$ of the vortex core in the nanodisk:

$$\begin{aligned} \dot{\theta} &= \frac{\kappa}{G}\left(1+\zeta s^2\right) \\ \dot{s} &= \frac{D_0 \kappa s}{G^2}\left(\frac{a_j I G}{D_0 \kappa \pi R^2} - 1 + (\zeta+\xi)s^2\right) \quad . \end{aligned}$$

The different parameters are summarized in table I, including their meaning and value chosen for the performed simulations. Furthermore, I is the applied dc current, and $\kappa(1+\zeta s^2)$ the confinement stiffness with κ its linear part and ζ its nonlinearity factor. In the table, we show the values of the magnetostatic confinement κ_{ms} with nonlinear part κ'_{ms}, and the Oersted field confinement κ_{Oe} with nonlinear part κ'_{Oe}. It is $\kappa = \kappa_{ms} + \kappa_{Oe} I/(\pi R^2)$ and $\zeta = \frac{\kappa'_{ms}+\kappa'_{Oe}I/(\pi R^2)}{\kappa_{ms}+\kappa_{Oe}I/(\pi R^2)}$.

$R = 187.5\,\mathrm{nm}$	nano-dot radius
$D_0 = 4.28 \cdot 10^{-15}\,\mathrm{kg.rad^{-1}.s^{-1}}$	linear damping coefficient
$\xi = 0.6$	nonlinear damping coefficient
$G = 2.0 \cdot 10^{-13}\,\mathrm{kg.rad^{-1}.s^{-1}}$	gyrovector amplitude
$a_j = 3.9 \cdot 10^{-16}\,\mathrm{kg.m^2.A^{-1}.s^{-2}}$	spin-transfer torque efficiency
$\kappa_{ms} = 4.05 \cdot 10^{-4}\,\mathrm{kg.s^{-2}}$	magnetostatic confinement
$\kappa'_{ms} = 1.01 \cdot 10^{-4}\,\mathrm{kg.s^{-2}}$	nonlinear magnetostatic confinement
$\kappa_{Oe} = 1.42 \cdot 10^{-15}\,\mathrm{kg.m^2.A^{-1}.s^{-2}}$	Oersted field confinement
$\kappa'_{Oe} = -7.12 \cdot 10^{-16}\,\mathrm{kg.m^2.A^{-1}.s^{-2}}$	nonlinear Oersted field confinement

Table I: Parameters used for the simulation of the vortex dynamics in presence of thermal and flicker noise.

Flicker noise generation

In order to introduce the flicker noise in the Thiele equation framework, we model a generating noise process of $1/f^1$ spectral shape, as shown in fig. 1 of the main text. This is then converted into the characteristic amplitude and phase noise PSD due to the dynamical nonlinear stochastic Langevin differential equations[4]. We define a random variable $r_{1/f}(t)$ that has a $1/f^1$ flicker noise PSD and is scaled by a factor λ. This random process is then added to the applied dc current I_{dc}:

$$I_{STO}(t) = I_{dc} + \lambda r_{1/f}(t) \quad . \tag{1}$$

As elaborated in the main text, $\lambda = 2.5 \cdot 10^{-4}$ was chosen in such a way that the final amplitude and phase noise PSD are meaningful and similar to those observed experimentally. In order to produce the random stream variable $r_{1/f}(t)$, we choose as a first step to construct a Fourier series decomposition with Fourier amplitude coefficients that have a $1/f$ decay and uniformly distributed random phase coefficients. By taking the inverse Fourier transform of this constructed decomposition, we obtain our $1/f$ random stream variable $r_{1/f}(t)$. As shown in fig. 1 of the main text, the noise PSD of the injected dc current $I_{STO}(t)$ in the simulation follows a $1/f$ relation for a broadband frequency bandwidth.

B. Discussion of the generating noise

The flicker generating noise, which leads to the $1/f^{\beta}$ power law characteristics in the amplitude and phase noise PSDs of the STVO, itself exhibits a $1/f^{1}$ spectral shape[4]. Its fundamental origin is not yet well understood, and in the main paper we model this original generating noise to be caused by the supplying dc source.

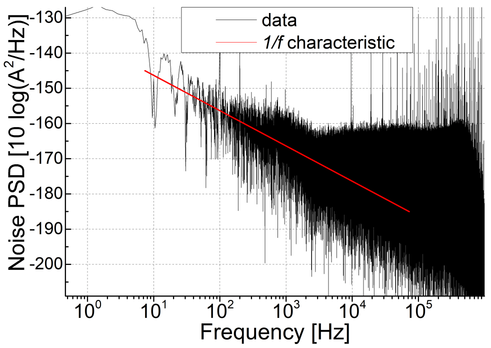

Figure 1: $1/f$ flicker noise characteristics of the supplying Keithley 6220 dc current source.

To gain more insight into the particular origins of the flicker noise and the influence of the current source characteristics, we can compare the noise PSD of the modelled generating noise (in the main paper) with the experimental noise PSD of the current source. In fig. 1, we present the measured noise characteristics of the used Keithley 6220 current source on the circuit with a $50\,\Omega$ resistance. The shown data are measured at $8\,\mathrm{mA}$. Indeed, the noise PSD does not change much within the current interval $]0;10]\,\mathrm{mA}$. Above, the current source changes range and the noise PSD might be different.

We see that indeed the noise PSD exhibits a $1/f$ spectral shape. Comparing the modelled generating noise with the measured noise PSD of the current supply, we find that the magnitude of the modelled noise is much higher than the one from the measurement, i.e. for instance at $10^4\,\mathrm{Hz} \sim -155\,\mathrm{dB}$ in the simulation compared to $\sim -180\,\mathrm{dB}$ for the measurement. We can conclude here that indeed different mechanisms contribute to the characteristic $1/f$ flicker noise. These might be fluctuations in the magnetic texture, the impact of defects and/or inhomogeneities in the magnetic layers or the tunnel barrier, or also external fluctuations of the applied magnetic field. Hence, also the noise of the dc current supply contributes but is not the major origin for the $1/f$ flicker noise in spin torque oscillators. However, in the simulation we model all these different origins of $1/f$ noise to be included in the dc current fluctuations. This approach is justified as the $1/f$ fluctuations mainly scale with the system power supply[4] and the current also comprises for effects in the spin torque, itself including the magnetic texture or the barrier as flicker noise origins.

C. Phase variance ↔ phase noise relation

Taking the Wiener-Khintchine theorem, the autocorrelation of the phase fluctuations $\delta\phi$ can be expressed through the phase noise PSD. Taking also the definition of the autocorrelation, we get:

$$K_{\delta\phi}(\tau) = \frac{1}{2\pi}\int_{-\infty}^{\infty} S_{\delta\phi}(\omega) e^{i\omega\tau} d\omega = \langle \delta\phi(t+\tau)\ \delta\phi(t)\rangle$$

We define the phase difference at times t and τ:

$$\psi(t,\tau) = \phi(t+\tau) - \phi(t) = \delta\phi(t+\tau) - \delta\phi(t)$$

Its variance function is:

$$\begin{aligned}\Delta\phi^2 &= \langle\psi^2\rangle - \langle\psi\rangle^2 = \langle[\delta\phi(t+\tau) - \delta\phi(t)]^2\rangle - 0\\ &= \langle\delta\phi(t+\tau)^2\rangle + \langle\delta\phi(t)^2\rangle - 2\,\langle\delta\phi(t+\tau)\delta\phi(t)\rangle\\ &= 2\left[\langle\delta\phi^2\rangle - \langle\delta\phi(t+\tau)\delta\phi(t)\rangle\right]\end{aligned}$$

This means that the variance function $\Delta\phi^2$ can be expressed in terms of phase noise:

$$\begin{aligned}\frac{\Delta\phi^2}{2} &= K_{\delta\phi}(0) - K_{\delta\phi}(\tau) = \frac{1}{2\pi}\int_{\mathbb{R}} \left(1 - e^{i\omega t}\right)\cdot S_{\delta\phi}(\omega) d\omega\\ &= \frac{1}{2\pi}\int_{\mathbb{R}} \left(1 - \cos(\omega t) - i\sin(\omega t)\right)\cdot S_{\delta\phi} d\omega\end{aligned}$$

Because $S_{\delta\phi}(\omega)$, similar to the autocorrelation function $K(t)$, is always an even function (Cauchy principal value), the expression can be simplified to:

$$\frac{\Delta\phi^2}{2} = \frac{1}{\pi}\int_{0}^{\infty} \left(1 - \cos(\omega t)\right)\cdot S_{\delta\phi} d\omega \quad . \tag{2}$$

D. Phase variance ↔ autocorrelation relation

Here, we discuss the autocorrelation function of the oscillation signal $c = \sqrt{p}e^{i\phi}$, not of the fluctuations as done above. The signal power is $p = p_0 + \delta p$ and the phase $\phi(t) = -\omega(p_0)t + \phi_i + \delta\phi(t)$. We assume the power fluctuations little compared to the phase fluctuations: $\delta p \ll \delta\phi$. It is for the autocorrelation:

$$\begin{aligned}K(\tau) &= \langle\sqrt{p_0+\delta p}\ e^{i\phi(t+\tau)}\sqrt{p_0+\delta p}\ e^{-i\phi(t)}\rangle\\ &\approx p_0\,\langle e^{i[\phi(t+\tau)-\phi(t)]}\rangle \approx p_0 e^{-i\omega(p_0)\tau}\,\langle e^{i[\delta\phi(t+\tau)-\delta\phi(t)]}\rangle\\ &= p_0 e^{-i\omega(p_0)\tau}\,\langle e^{i\psi(t,\tau)}\rangle\end{aligned}$$

As defined already above, we used $\psi(t,\tau) = \delta\phi(t+\tau) - \delta\phi(t)$.

Because the fluctuations are defined by a stationary ergodic process following a 0-centered Gaussian law, ψ has the following probability function:

$$p(\psi) = \frac{1}{\Delta\phi\sqrt{2\pi}} e^{-\frac{\psi^2}{2\Delta\phi^2}} \quad .$$

Using this, it follows:

$$\langle e^{i\psi(t,\tau)}\rangle = \int\limits_{-\infty}^{\infty} e^{i\psi} p(\psi) d\psi$$

$$= \int\limits_{-\infty}^{\infty} \left[\cos(\psi) + \underbrace{i\sin(\psi)}_{\text{antisymmetric}\rightarrow=0} \right] p(\psi) d\psi$$

$$= \frac{1}{\Delta\phi\sqrt{2\pi}} \int\limits_{-\infty}^{\infty} \cos(\psi) e^{-\psi^2/(2\Delta\phi^2)} d\psi = e^{-\Delta\phi^2/2} \quad .$$

In the last step, the Gaussian integral was evaluated.

In total, we get the final result:

$$K(\tau) \approx p_0 e^{-i\omega(p_0)\tau} e^{-\Delta\phi^2/2} \quad .$$

* steffen.wittrock@cnrs-thales.fr
† vincent.cros@cnrs-thales.fr
[1] A. A. Thiele, Phys. Rev. Lett. **30**, 230 (1973).
[2] A. Dussaux, A. V. Khvalkovskiy, P. Bortolotti, J. Grollier, V. Cros, and A. Fert, Physical Review B **86** (2012), 10.1103/physrevb.86.014402.
[3] E. Grimaldi, A. Dussaux, P. Bortolotti, J. Grollier, G. Pillet, A. Fukushima, H. Kubota, K. Yakushiji, S. Yuasa, and V. Cros, Phys. Rev. B **89**, 104404 (2014).
[4] S. Wittrock, S. Tsunegi, K. Yakushiji, A. Fukushima, H. Kubota, P. Bortolotti, U. Ebels, S. Yuasa, G. Cibiel, S. Galliou, E. Rubiola, and V. Cros, Physical Review B **99** (2019), 10.1103/physrevb.99.235135.
